# Increased Risk of Atopic Dermatitis in Preschool Children with Kawasaki Disease: A Population-Based Study in Taiwan

**DOI:** 10.1155/2013/605123

**Published:** 2013-08-27

**Authors:** Peng Yeong Woon, Wei Chiao Chang, Chi-Cheng Liang, Chun Hung Hsu, Sukhontip Klahan, Ying-Hsien Huang, Wei-Pin Chang, Ho-Chang Kuo

**Affiliations:** ^1^Department of Molecular Biology and Human Genetics, Tzu Chi University, Hualien 97004, Taiwan; ^2^Department of Clinical Pharmacy, Taipei Medical University, Taipei 110, Taiwan; ^3^Master Program for Clinical Pharmacogenomics and Pharmacoproteomics, School of Pharmacy, Taipei Medical University, Taipei 110, Taiwan; ^4^Department of Pharmacy, Taipei Medical University-Wanfang Hospital, Taipei 110, Taiwan; ^5^Cancer Center, Kaohsiung Medical University Hospital, Kaohsiung Medical University, Kaohsiung 807, Taiwan; ^6^Division of Emergency and Trauma Surgery, Department of Surgery, Kaohsiung Chang Gung Memorial Hospital and Chang Gung University, College of Medicine, Kaohsiung 83301, Taiwan; ^7^Department of Healthcare Management, Yuanpei University, Hsinchu 30015, Taiwan; ^8^Department of Pediatrics, Kaohsiung Chang Gung Memorial Hospital and Chang Gung University College of Medicine, Kaohsiung 83301, Taiwan

## Abstract

Kawasaki disease (KD) is an acute febrile systemic vasculitis and has been reported to be associated with allergic disease. The risk of atopic dermatitis (AD) in preschool children with KD has not been investigated. The study was to determine the longitudinal risk of the development of AD in preschool children with KD. A nationwide 5-year population-based study was performed using data from the National Health Insurance Database in Taiwan between 1999 and 2003. The risk factors for AD were compared between the 2 study groups during the follow-up period using the Cox proportional hazards model. In addition, plasma interleukin (IL)-5 levels were analyzed in normal subjects and KD patients. Among the 1440 subjects included, 21.6% developed AD during the 5-year follow-up period, of which 30.3% and 18.7% belonged to the study cohort and the comparison group, respectively. Children with KD were 1.25 times more likely to have AD than those in controls (*P* = 0.04). Levels of IL-5 and IgE were significantly higher in KD patients. Children with KD had a higher risk of developing AD during the 5-year follow-up period than the control group. Increased IL-5 and IgE levels may be key factors contributing to the risk of AD.

## 1. Introduction

Kawasaki disease (KD) is an acute febrile systemic vasculitis that was first described by Kawasaki et al. [1]. In developed countries, it is the leading cause of acquired heart disease in children. However, the etiology of KD remains unknown [2–5]. The acute stage of KD is characterized by systemic inflammatory changes, including high-grade fever lasting ≥5 days, bilateral conjunctivitis, oral mucosal changes, fissured lips, unilateral cervical lymphadenopathy (>1.5 cm), dysmorphic skin rash, and erythema of the palms and soles [6, 7]. The most serious complication of KD is the involvement of coronary artery lesions (CALs), including myocardial infarction, coronary artery fistula formation [8], coronary artery dilatation, and coronary artery aneurysm (CAA) [9, 10]. Children <5 years of age are the most susceptible population. According to recent epidemiologic studies, the incidence of KD is significantly higher in Asian populations. Japan has the highest annual incidence rates of KD in the world [11], followed by Korea [12] and Taiwan [13] (239, 113, and 69 per 100,000 children <5 years of age, resp.). The incidence of KD has increased globally in recent years [10, 14–17].

The prevalence of allergic diseases, including asthma, atopic dermatitis (AD), and allergic rhinitis, has increased worldwide in recent decades, primarily in developed countries [18–21]. This increase has been attributed to environmental factors, including improved hygiene, increased use of antimicrobial medications, consumption of sterile foods, and reduced family size, which have resulted in low rates of infection during childhood and less contact with microbes. Burns et al. [22] reported that the associations of KD with AD, elevated serum IgE levels, eosinophilia, and increased circulating numbers of monocyte/macrophages expressing the low-affinity IgE receptor may be related to the effects of IL-4. Brosius et al. [23] showed that the incidence of AD among children with KD was 9 times greater than that among control subjects. Liew et al. [24] reported that KD may be a risk factor for subsequent allergic disease and postulated that KD occurs more frequently in children at risk of immune disequilibrium, with an initial abnormal inflammatory response and a subsequent increase in allergic manifestations. We previously reported that the T helper (Th) type 2 immune response was elevated in the acute stage of KD, and the involvement of eosinophils [25], interleukin-4 (IL-4), IL-5 [26], and eotaxin was shown. The changes in the levels of eosinophils correlated with changes in the levels of IL-5 but not eosinophil cationic protein (ECP), suggesting the involvement of a Th2 immune reaction in KD. We also reported that genes involved in immune regulation (CTLA-4 and TGF-beta signaling pathway) were associated with KD with or without CAL formation [27, 28]. Importantly, both CTLA-4 and TGF-beta signaling pathways have been shown to be associated with the development of AD [29–31]. Indeed, an abnormal Th1/Th2 balance plays a key role in the development of KD [7, 25, 26, 32–35]. However, the association between preschool children with KD and AD has not been investigated to date. 

The incidence of KD has increased [17, 36] in parallel with the increase in the incidence of allergic diseases worldwide [37]. In the present study, we examined the incidence of AD in patients with KD during a 5-year follow-up period using a nationwide population-based dataset obtained from Taiwan's National Health Insurance (NHI) health care database. We also analyzed the levels of IL-5, an important biomarker of allergic diseases, and detected high levels of IL-5 expression in KD patients during the acute stage of the disease.

## 2. Methods

### 2.1. Database

The present study used data obtained from Taiwan's NHI database. Taiwan began the NHI program in 1995 as a mandatory plan that covered nearly 99% of the Taiwanese population of almost 23 million residents. The NHI program was organized by Taiwan's Bureau of National Health Insurance (BNHI). To encourage researchers to perform health studies on issues relevant to the NHI program, the BNHI created and released the Longitudinal Health Insurance Database 2005 (LHID2005) to the public for research purposes.

Our study adopted the LHID2005, which included registration and medical claims for 1 million randomly sampled individuals from all the NHI enrollees (*n* = 23 million). The dataset included information on all medical claims of these 1 million beneficiaries between 1996 and 2010. The National Health Research Institute (NHRI) claims that there were no statistically significant differences in age, sex, or health care costs between the selected group and all enrollees. Because the LHID2005 dataset was established for research purposes, personal identifying information could not be obtained. As the NHRI had addressed the confidentiality assurance issue, the present study was exempted from full review by the Institutional Review Board.

### 2.2. Study Population

A total of 360 preschool KD children were considered eligible for the study. The inclusion criteria were age ≤6 years and newly diagnosed KD (ICD-9-Code 446.1) between January 1999 and December 2003. Moreover, to ensure the diagnostic precision of the administrative dataset, we only included preschool children who had been diagnosed with KD ≥2 times during outpatient visits or ≥1 time as inpatients. We excluded patients diagnosed with KD before 1999 (*n* = 43). Our study assigned each child a first diagnosis of KD as the index date between January 1999 and December 2003. Control groups were randomly selected from the remaining subjects of the LHID2005 and matched with a control-to-case ratio of 3 : 1 on the basis of age, sex, and index year.

Each child was tracked for 5 years from the index date to determine the incidence of AD (ICD-9-CM 691.8, 692.9). To improve diagnosis accuracy, the selection criteria required that all AD cases ICD-9 code was assigned by pediatrician or dermatologist. In addition, to investigate the relationship between AD and particular comorbidities, we analyzed certain covariables such as chronic obstructive pulmonary disease (COPD; ICD-9-CM 490.X-496.X), hyperlipidemia (ICD-9-CM 272.X), asthma (ICD-9-CM 493.X), allergic rhinitis (ICD-9-CM 477.X), and heart disease (ICD-9-CM 410.X-414.X) in our cohort study.

### 2.3. Level of Urbanization and Geographic Region

Regarding urbanization level, the Taiwanese National Health Research Institute performed cluster analysis to study urbanization levels, which resulted in the division of 359 towns or cities in Taiwan into 7 categories, with 1 representing “the most urbanized” and 7 representing “the least urbanized.” These 7 categories were defined by incorporating various variables such as population density, proportion of elderly people and agriculture workers, number of physicians per 100,000 people, and different educational levels, which were based on the 2000 Taiwan census data. Only a small number of preschool children with KD were identified in levels 5, 6, and 7. Therefore, these 3 levels were combined into a single group. For the purposes of the study, we divided Taiwan into 4 geographic regions, northern, central, southern, and eastern Taiwan. Urbanization level and geographic region were incorporated into our model to adjust for possible differences in access to medical care.

### 2.4. Measurement of Interleukin-5 and IgE Levels

This study was approved by the Institutional Review Board of Chang Gung Memorial Hospital. Blood samples were collected after obtaining informed consent from the parents or guardians. Blood samples collected before intravenous immunoglobulin (IVIG) treatment (within 24 hours before IVIG treatment, acute stage) and after IVIG treatment (at least 3 weeks after IVIG treatment, subacute stage) were included in the analysis.

Plasma levels of IL-5 were assessed using the Upstate Beadlyte Human Cytokine Beadmates system (Upstate Group, Inc.) in 64 patients with KD patients and 14 controls, according to a modification of the method described previously [26]. In brief, 50 *μ*L of plasma was mixed with multiplexed antibody-conjugated beads and subjected to multi-channel detection of the bead array. Acquired fluorescence data were assessed using the MasterPlex TM QT software (Ver. 1.2; MiraiBio, Inc.). Cytokine concentrations were calibrated by interpolation of a series of well-known standard samples following the manufacturer's recommendations. The assay sensitivity of these cytokines was 0.2 pg/mL. To avoid interassay bias in the immunoassays, the cytokines in paired samples before and after IVIG therapy were measured at the same time. IgE levels were quantified by a sensitive, IgE detection system with the automatic Pharmacia UnicAP device (Pharmacia & Upjohn Diagnostics AB, Uppsala, Sweden) using a fluorescence enzyme immunoassay system.

### 2.5. Statistical Analysis

The SPSS (Statistical Package for Social Science) statistical software version 18.0 (SPSS, Inc.) was used for data analysis. All data were expressed as frequency and percentage or mean and standard deviation. Chi-square test was performed to assess differences in geographic location and urbanization level of patients' residences between the study group and comparison group. Stratified Cox proportional hazard regression analysis (stratified by sex, age group, and year of index) was performed to investigate the risk of subsequent AD during the 5-year follow-up period in preschool children with and without KD. All subjects were followed from the index date until the detection of AD or the end of the 5-year follow-up period. Hazard ratios (HRs) along with 95% confidence intervals (CIs) were calculated to determine the risk of colorectal cancer. Plasma levels of IL-5 were analyzed by the Mann-Whitney *U* test. A 2-sided *P* value <0.05 was considered statistically significant.

## 3. Results

### 3.1. Correlation between KD and AD

**Figure 1:**
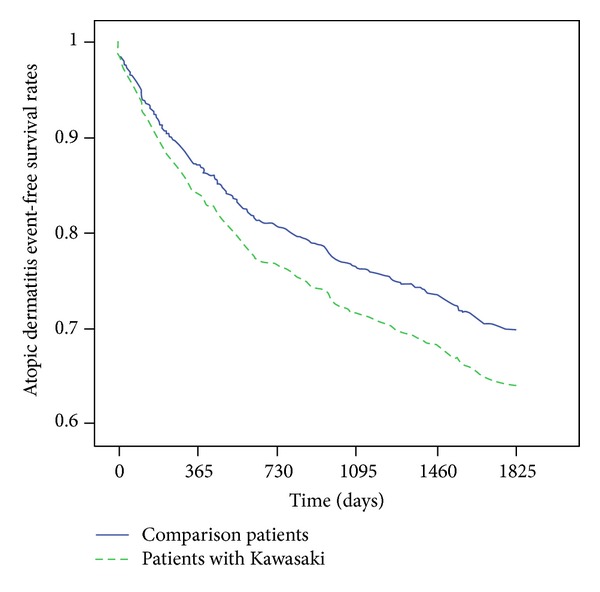
Event free survival rates of atopic dermatitis for children with Kawasaki disease and the comparison group between 1999 and 2003.

A total of 360 preschool children diagnosed with KD matched the inclusion criteria, and 1080 preschool children were included in the comparison cohort.  Table 1 shows the demographic characteristics of the study subjects stratified by the presence of KD. Diseases such as COPD, hyperlipidemia, asthma, allergic rhinitis, and heart disease were associated with KD.  Table 2 shows that among the 1440 patients comprising the study population, AD was detected in 311 subjects during the follow-up period. AD was detected in 109 children with KD, representing an incidence rate of 104.68 per 1000 person years, and in 202 subjects in the comparison cohort, representing an incidence rate of 76.04 per 1000 person years. Cox regression analysis also showed that the crude HR of AD was 1.43 times greater for children with KD than for the comparison cohort (95% CI = 1.11−1.85; *P* < 0.01). The HR remained significant after adjusting for potential confounders (adjusted HR: 1.25, 95% CI = 1.01−1.54; *P* < 0.05). The results of survival analysis with the Cox regression model are shown in Figure 1.

### 3.2. Expression Levels of IL-5 and IgE Were Significantly Higher in KD Patients than Normal Subjects

**Figure 2:**
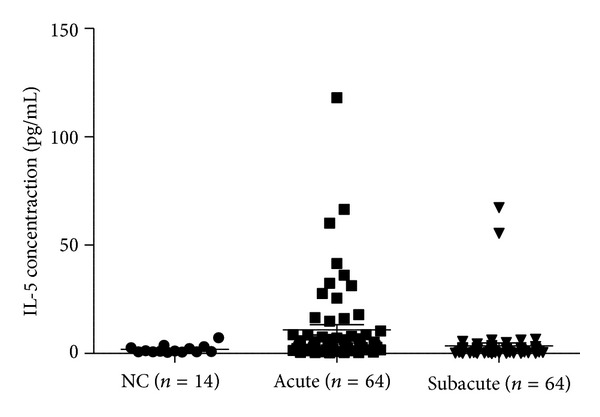
Plasma levels of interleukin-5 (IL-5) were detected in normal controls (*n* = 14), patients with acute KD (*n* = 64, before IVIG treatment), and subacute KD (*n* = 64, at least 3 weeks after IVIG treatment). Patients with acute KD showed significantly higher levels of IL-5 than normal controls.

**Figure 3:**
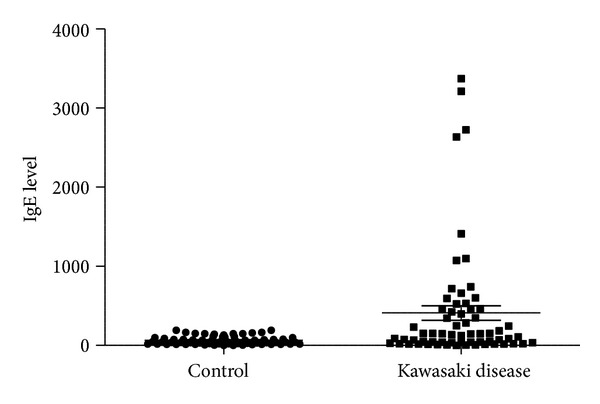
Expression levels of IgE were detected in normal controls (*n* = 64) and patients with KD (*n* = 64). Patients with KD showed significantly higher levels of IgE than normal controls.

Plasma levels of IL-5 before IVIG treatment were significantly higher in KD patients (*n* = 64) than those in the controls (*n* = 14) (*P* = 0.001). Moreover, IL-5 levels were significantly lower in patients in the subacute stage of KD (*n* = 64) than those in patients in the acute stage of KD (*P* < 0.001) (Figure 2). We further tested the expression levels of IgE in KD patients and control subjects. As shown in Figure 3, high IgE expression level in KD patients was observed (*P* = 0.0003).

## 4. Discussion

This is the first population-based study to use a nationwide dataset to investigate the relation between AD and KD in children during a 5-year follow-up period. Our results indicated that children with KD have a higher risk of developing AD than healthy controls (95% CI = 1.01–1.54; *P* < 0.05).

KD is a systemic vasculitis of unknown etiology, and its immunopathogenesis remains unclear. Several lines of evidence have shown an imbalance between Th1 and Th2 immune reactions in KD patients. Th1 immune related responses (IFN-gamma, tumor necrosis factor-alpha, IL-1*β*, and IL-10) [38, 39] and Th2 immune related responses (eosinophils, IL-4 [22], IL-5 [25, 26], eotaxin, and total IgE) have been reported to be associated with the susceptibility to KD and disease outcomes. Furthermore, serum IgE levels were shown to be increased in KD patients during the acute stage of the disease [34]. Therefore, an association between KD and allergic diseases has been suggested.

IL-5 and IL-5 receptor alpha polymorphisms were reported to be associated with AD [40]. IL-5 and Th2 cells promote AD development mediated by CCL8 [41]. Elevated numbers of blood and tissue eosinophils are present in allergic diseases and KD [25], suggesting that eosinophils play an important pathogenic role in these diseases. The regulation of eosinophil maturation, recruitment, and survival is controlled by a group of factors, including IL-5. Therefore, IL-5 has been proposed as a potential molecular target in the treatment of allergic diseases [42]. In the present study, IL-5 was found to be expressed at higher levels in KD patients in the early stage of KD. IVIG treatment resulted in a significant decrease in IL-5 levels. Recent studies indicated that keratinocyte TSLP and IL-33 are generated by the damaged AD barrier and trigger Th2 responses [43, 44]. Thus, this same mechanism may account for increased Th2 responses during the early phases of KD.

The molecular mechanisms common to AD and KD are still unclear. Previous genetic polymorphism studies in Japanese and Taiwanese populations indicated that genetic variants of the *ORAI1 *gene, which encodes a membrane calcium channel subunit, contribute to the susceptibility to atopic dermatitis [45]. Although an association between genetic polymorphisms of *ORAI1 *and the risk of KD was not observed in a Taiwanese population study [46], inositol 1,4,5-trisphosphate 3-kinase C (ITPKC) gene, a gene upstream of *ORAI1*, was shown to be an important biomarker for the susceptibility to KD [10]. The polymorphism of *ITPKC *(rs28493229) altered the transcriptional level of the mature mRNA by interfering with the RNA splicing efficiency [47]. ITPKC protein phosphorylates inositol 1,4,5-trisphosphate (IP_3_), which plays a role in ORAI1-mediated immune responses [39]. Thus, ITPKC-mediated calcium signaling may be a common molecular mechanism underlying KD and AD.

The incidence of AD among children with KD has been reported to be greater than that of control subjects and was associated with higher serum IgE concentrations [19]. Indeed, we also observed high-expression level of IgE in KD patients in this study. Another study that included Japanese children reported a higher incidence of AD in children with KD than in healthy controls, even among children with no family history of allergies [33]. In addition, KD may be a risk factor for subsequent allergic disease and therefore may be associated with allergic manifestations [20]. Consistent with the results of previous studies, our results indicated that children with KD were more likely to develop AD than healthy children.

Our study had few limitations. First, certain factors such as the incidence of household pets, cigarette smoking in the family, or the family history of allergy, which could affect the susceptibility to the development of AD in children, were not included in the analysis. Second, household income and economic status are other indexes besides urbanization or geographic region that could be important for the evaluation of medical access or medical utilization. Third, the expression levels of IL-5 and IgE in AD patients should be evaluated to further confirm the association between AD and KD in the Taiwanese pediatric population. Despite these limitations, the use of a nationwide dataset, which provided a large sample size, and adjustment for certain covariates related to KD enabled the detection of a significant relation between KD in preschool children and the risk of developing AD during the 5-year follow-up period.

## 5. Conclusion

In the present study, we showed that KD is associated with the risk of AD in preschool children, although the underlying mechanisms remain unclear. Increased levels of IL-5 and IgE may be key factors contributing to the risk of AD. Further studies are required to verify our results and to include related physiological variables.

## Figures and Tables

**Table 1:** Demographic characteristics of the study groups, stratified by the presence or absence of Kawasaki disease between 1999 and 2003 (*n* = 1440).

	Patients with Kawasaki disease (*n* = 360)		Patients without Kawasaki disease (*n* = 1080)		*P* value
	*n*	Percentage	*n*	Percentage	
Gender					1
Male	194	53.9	582	53.9	
Female	166	46.1	498	46.1	
Age (years)					1
0	59	16.4	177	16.4	
1	121	33.6	361	33.4	
2	67	18.6	203	18.8	
3	38	10.6	113	10.5	
4	37	10.3	112	10.4	
5	20	5.6	62	5.7	
6	18	5.0	52	4.8	
Urbanization level					0.03
1 (most urbanized)	111	30.8	257	23.8	
2	128	35.6	384	35.6	
3	51	14.2	197	18.2	
4 (least urbanized)	70	19.4	242	22.4	
Geographic region					0.41
Northern	192	53.3	526	48.7	
Central	96	26.7	304	28.1	
Southern	64	17.8	216	20.0	
Eastern	8	2.2	34	3.1	
COPD					<0.001
Yes	212	58.9	503	46.6	
No	148	41.1	577	53.4	
Hyperlipidemia					0.01
Yes	6	1.7	4	0.4	
No	354	98.3	1076	99.6	
Asthma					<0.001
Yes	177	49.2	405	37.5	
No	183	50.8	675	62.5	
Allergic rhinitis					<0.001
Yes	243	67.5	612	56.7	
No	117	32.5	468	43.3	
Heart disease					<0.001
Yes	53	14.7	29	2.7	
No	307	85.3	1051	97.3	

**Table 2:** Hazard ratios (HRs) of atopic dermatitis among patients with Kawasaki disease during a 5-year follow-up period from the first ambulatory visit or inpatient care between 1999 and 2003.

Development of atopic dermatitis	Total		Patients with Kawasaki disease		Patients without Kawasaki disease	
	No.	(Percentage)	No.	(Percentage)	No.	(Percentage)
Five-year follow-up period						
Yes	311	21.6	109	30.3	202	18.7
No	1129	78.4	251	69.7	878	81.3
Crude HR (95% CI)			1.43 (1.11–1.85)**		1	
Adjusted HR (95% CI)			1.25 (1.01–1.54)*		1	

Total sample number = 1440.

Crude and adjusted HRs were calculated using Cox proportional hazard regression.

Adjustments were made for patients' urbanization level, geographic region, COPD, hyperlipidemia, asthma, allergic rhinitis, and heart disease.

*Indicates that *P* < 0.05; **indicates that *P* < 0.01.
